# Reciprocating *vs* Rotary Instrumentation in Pediatric Endodontics: Cone Beam Computed Tomographic Analysis of Deciduous Root Canals using Two Single-file Systems

**DOI:** 10.5005/jp-journals-10005-1332

**Published:** 2016-04-22

**Authors:** Attiguppe R Prabhakar, Chandrashekar Yavagal, Kratika Dixit, Saraswathi V Naik

**Affiliations:** 1 Professor and Head, Department of Pedodontics and Preventive Dentistry, Bapuji Dental College and Hospital, Davangere, Karnataka, India; 2 Reader, Department of Pedodontics and Preventive Dentistry, Bapuji Dental College and Hospital, Davangere, Karnataka, India; 3 Postgraduate Student, Department of Pedodontics and Preventive Dentistry, Bapuji Dental College and Hospital, Davangere, Karnataka, India; 4 Reader, Department of Pedodontics and Preventive Dentistry, Bapuji Dental College and Hospital, Davangere, Karnataka, India

**Keywords:** Cone beam computed tomography, One shape, Pulpectomy, Reciprocating motion, Wave one.

## Abstract

**Background:** Primary root canals are considered to be most challenging due to their complex anatomy. "Wave one" and "one shape" are single-file systems with reciprocating and rotary motion respectively. The aim of this study was to evaluate and compare dentin thickness, centering ability, canal transportation, and instrumentation time of wave one and one shape files in primary root canals using a cone beam computed tomographic (CBCT) analysis.

**Study design:** This is an experimental, *in vitro* study comparing the two groups.

**Materials and methods:** A total of 24 extracted human primary teeth with minimum 7 mm root length were included in the study. Cone beam computed tomographic images were taken before and after the instrumentation for each group. Dentin thickness, centering ability, canal transportation, and instrumentation times were evaluated for each group.

**Results:** A significant difference was found in instrumentation time and canal transportation measures between the two groups. Wave one showed less canal transportation as compared with one shape, and the mean instrumentation time of wave one was significantly less than one shape.

**Conclusion:** Reciprocating single-file systems was found to be faster with much less procedural errors and can hence be recommended for shaping the root canals of primary teeth.

**How to cite this article:** Prabhakar AR, Yavagal C, Dixit K, Naik SV. Reciprocating *vs* Rotary Instrumentation in Pediatric Endodontics: Cone Beam Computed Tomographic Analysis of Deciduous Root Canals using Two Single-File Systems. Int J Clin Pediatr Dent 2016;9(1):45-49.

## INTRODUCTION

Pulpectomy is the intervention of choice for treating infected pulp tissues in pediatric dental patients.^1^ Earlier this treatment was performed with hand instruments. Rotary instruments were introduced to pediatric endodontics by Barr et al^2^ in the year 2000. However, morphological challenges posed by the primary root canals demand an improvement in the quality and design of contemporary rotary instruments so as to avoid undesirable complications, *viz.,* canal transportation, ledges, strip perforations, zips, etc.^1^

Nickel titanium (NiTi) was developed 40 years ago at the Naval Ordnance Laboratory of Silver Springs, Maryland.^3^ Rotary NiTi files follow the original anatomy of curved canals in primary teeth, and hence minimize the risk of procedural errors.^4^ Also, a funnel-shaped canal preparation is obtained, and thus a uniform and more predictable obturation with pastes can be achieved.^2^ More importantly, rotary files shorten the preparation time considerably and, therefore, suit the shorter attention spans of children, thus, increasing their cooperation for the endodontic procedure.^5^

In the bygone decade, several rotary NiTi endodontic file systems have been launched to improve the shaping procedure. However, all these systems recommended the use of a series of files to accomplish the final shape. Recently, the concept of single-file systems has been introduced and is currently being debated for its applicability in contemporary endodontics.^6^ Two such single-file systems are wave one and one shape employing reciprocating and rotatory motions respectively. Reciprocating motion is basically any back or forth motion, in clockwise and anticlockwise direction. The main advantage of such a motion is the reduction in the number of endodontic mishaps through instrument separation, which is primarily due to avoidance of continuous dentinal over engagement. The added advantages of these single-file systems include reduction in the working time, prevention of cross-contamination, and improved safety of the shaping procedures.^6^ All these factors assume a greater importance while we treat children.

Few recent studies on these newer instruments have demonstrated an excellent shaping and centering ability.^7,8^ However, these studies were conducted on permanent teeth. Hence, there is a need to assess their efficacy in primary teeth, which are anatomically more challenging than the permanent teeth. Hence, the present study was planned to evaluate and compare centering ability, computerized tomography (CT), dentin thickness, and instrumentation time of wave one and one shape single-file systems on primary teeth.

## MATERIALS AND METHODS

A total of 24 freshly extracted human primary teeth (16 molars, 6 incisors, 2 canines) collected from the Department of Pedodontics and Preventive Dentistry, Bapuji Dental College and Hospital, Davangere, Karnataka, were included in the study. Institutional ethical board approval was obtained prior to the study. Groups that were used in the study:

*Group* 1: Wave one single-file system

*Group* 2: One shape single-file system.

### Sample Preparation

The primary teeth with minimum 7 mm root length were included in the study.^9^ The specimens were embedded in autopolymerizing acrylic resin using a plastic mold. In order to prevent the resin from entering and polymerizing into the apical foramen, the apices of the roots were sealed with wax. Acrylic resin was mixed according to the manufacturer’s instructions and poured into the mold. Each sample was inserted into the unset acrylic resin so that its long axis was parallel to the long axis of the mold to ensure standardization of the specimens for tomographic imaging.^10^

### Root Canal Preparation

The canals were first scouted with a #10 K-file to check patency and to precisely determine the working length. A glide path was established before instrumentation. Before shaping, a drop of ethylenediaminetetraacetic acid (Well-prep; Vericom Co Ltd., Korea) was placed inside the coronal reservoir for lubrication. The preparation was performed with single-file systems, wave one (primary) (Dentsply maillefer, Switzerland) and one shape (Micromega, France), in respective groups using X-smart plus endodontic motor (Dentsply Maillefer, Switzerland). As recommended by the manufacturers, speed of 400 rpm was used for one shape system. Sodium hypochlorite (1%) was used as irrigant during the procedure.

Cone Beam Computed Tomography

**Fig. 1:**
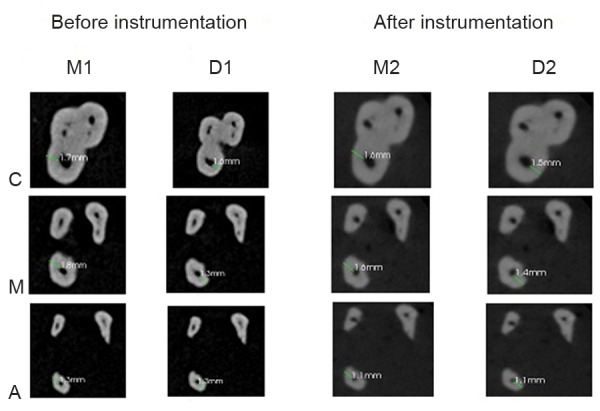
Dentin thickness for wave one (C: Cervical level, M: Middle level, A: Apical level)

**Fig. 2:**
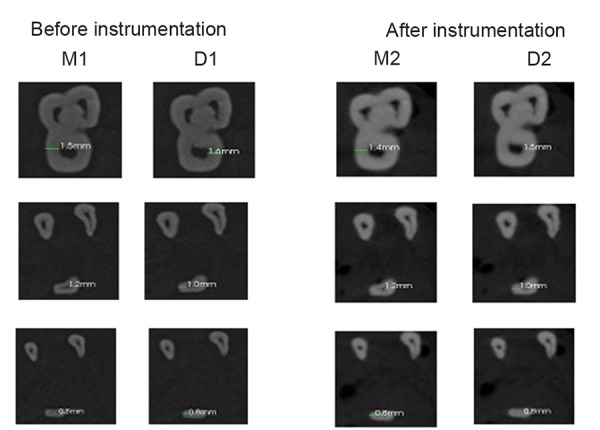
Dentin thickness for one shape

Teeth were scanned before and after mechanical preparation with Cone Beam Computed Tomography (CBCT) scanner (CS9300, Carestream Health Inc, Rochester, New York, USA) with the following parameters: 84 kVp, 5 mA, exposure time 20 seconds, field of view 50 mm. Sections were taken at coronal, middle and apical levels. The measurements of the noninstrumented areas and the measurements after root canal preparation were done voxel by voxel. M1 was the measurement of the quantity of voxels from the external surface of the mesial portion of the root to the mesial wall of the noninstrumented canal. M2 was the measurement of the quantity of voxels from the external root surface of the mesial portion of the root to the wall of the canal after instrumentation. D1 was the measurement of the quantity of voxels of the external surface of the distal portion of the root to the distal wall of the noninstrumented canal. D2 was the measurement of the quantity of voxels from the external surface of the distal portion of the root to the distal surface of the canal after instrumentation (Figs 1 and 2).^10^

Canal transportation was calculated from the following equation:

Canal transportation = (M1 - M2) - (D1 - D2)

Regarding transportation direction, CT equal to 0 (zero) meant lack of transportation, a negative value represented transportation to the distal direction, and a positive value represented transportation toward the mesial direction.

Centering ability ratio was calculated using the values obtained during the measurement of transportation using the following equation:

Centralization ability ratio = (M1 - M2)/(D1 - D2)

A result equal to 1.0 indicated perfect centralization. When this value was closer to zero, it implied that the instrument had a lower capacity to maintain itself in the central axis of the canal.^10^

Dentin thickness was measured on the axial cuts from the periphery of the pulp space to the outer surface of the tooth at three levels (cervical, middle, and apical).^10^

Instrumentation time (instrumentation plus irrigation) was measured using a digital chronometer.

## RESULTS

- Intergroup comparison for centering ability, CT, and dentin thickness was done by Kruskal-Wallis test.
- Mann-Whitney U-test was used for pairwise comparisons if Kruskal-Wallis was significant.
- Student’s t-test was used for instrumentation time comparison between the two groups.
- Statistical analysis was done using Statistical Packages for the Social Sciences (SPSS) version 16.

Dentin Thickness

**Graph 1:**
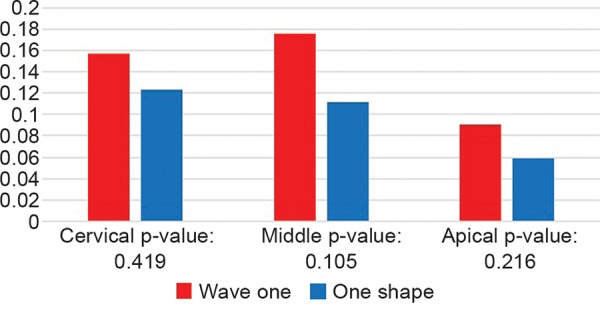
Dentin thickness

Statistical analysis showed no significant difference between wave one and one shape at any level (Table 1 and Graph 1).

Canal Transportation

**Graph 2:**
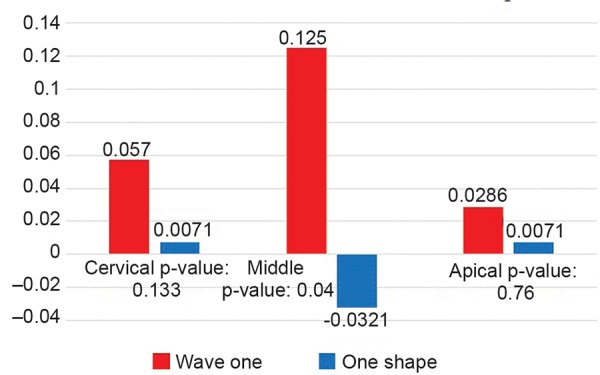
Canal transportation

Statistical analysis showed a significant difference in the measures at the middle level of the canal and wave one showed less CT as compared with one shape (Table 2 and Graph 2).

Centering Ability Ratio

**Graph 3:**
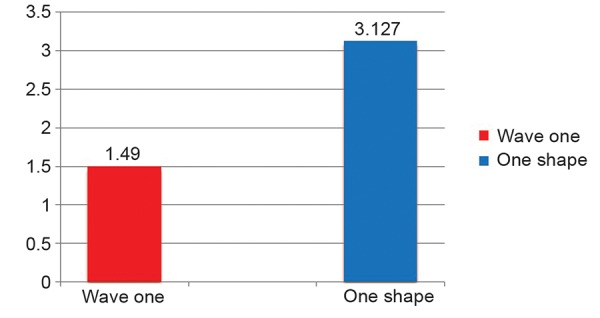
Centering ratio

Statistical analysis showed no significant difference between wave one and one shape at any level (Table 3 and Graph 3).

Instrumentation Time

**Graph 4:**
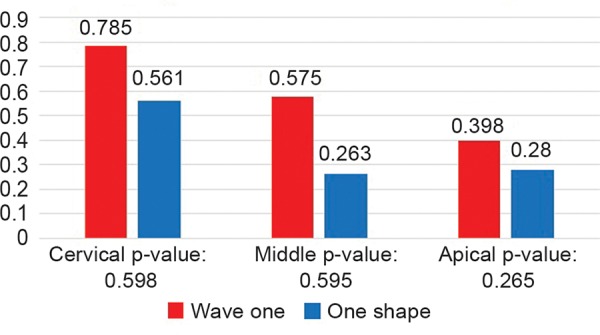
Instrumentation time

A significant difference in the instrumentation time of wave one and one shape was found. The mean instrumentation time of wave one was 1.49 minutes compared with that of one shape which was 3.127 minutes (Graph 4).

**Table 1:** **Table 1:** Dentin thickness

*Level*		*Wave one* *Mean ± SD (n = 28)*		*One shape* *Mean ± SD (n = 28)*		*p-value* *(n = 28)*	
Cervical		0.157 ± 0.139		0.123 ± 0.199		0.419	
Middle		0.176 ± 0.206		0.112 ± 0.219		0.105	
Apical		0.091 ± 0.085		0.0589 ± 0.154		0.216	

SD: Standard deviation

**Table 2:** **Table 2:** Canal transportation

*Level*		*Wave one* *Mean ± SD (n = 28)*		*One shape* *Mean ± SD (n = 28)*		*p-value* *(n = 28)*	
Cervical		0.057 ± 0.131		0.0071 ± 0.161		0.133	
Middle		0.125 ± 0.177		–0.0321 ± 0.272		0.004*	
Apical		0.0286 ± 0.165		0.0071 ± 0.235		0.760	

SD: Standard deviation; *p-value of 0.004 is <0.05, which confirms that there is a significant difference in canal transportation measures at middle level of canals

**Table 3:** **Table 3:** Centering ratio

*Level*		*Wave one* *Mean ± SD (n = 28)*		*One shape* *Mean ± SD (n = 28)*		*p-value* *(n = 28)*	
Cervical		0.785 ± 1.102		0.561 ± 0.955		0.598	
Middle		0.575 ± 0.866		0.263 ± 1.267		0.595	
Apical		0.398 ± 0.691		0.280 ± 1.100		0.265	

SD: Standard deviation

## DISCUSSION

Primary root canals are considered to be anatomically more complex and challenging compared with the permanent ones. This study was aimed to simplify the shaping procedure of primary canals by reducing the number of files as well as the amount of time taken.

Recently, a new wave one NiTi single-file reciprocating system has been introduced for better and simple root canal preparation. Only one single-shaping file is required to instrument the canal to an adequate size and taper. The main characteristics of this system are single use, a reciprocating action, and M-wire alloy manufacturing technology which improves strength and resistance to cyclic fatigue. Even more recently, one shape file has been introduced in which complete canal shaping is possible with only one single file, which is used in continuous rotation. The major advantages of this system are its economic viability, unique design, and presterilized usage with zero cross-contamination.

### Rotary *vs* Reciprocating Motion

The principles of rotary motion have been in clinical usage for close to 20 years now. However, the physics behind reciprocating motion is based on the "law of action and reaction," which results in a balanced force during canal instrumentation, as theorized by Roane et al.^11^ The reciprocating movement minimizes torsional and flexural stresses, increases the canal centering ability, and reduces the taper lock of the instrument within the canal. Recent studies have shown that an alternating reciprocating movement is a valid option to optimize endodontic instrumentation by reducing the risk of instrument fracture and root canal deformity.^12^ The use of such a reciprocating motion in place of continuous rotation could thus prove to be advantageous in terms of reducing the stress as well as the time required for the preparation of curved root canals,^13^ which are frequently encountered in pediatric endodontics.

In the present study, teeth with minimum 7 mm of root length were selected so as to simulate a clinical pulpectomy procedure where at least two-thirds of root length is considered to be necessary. Though a number of methods have been used to evaluate canal shape before and after instrumentation, CBCT imaging was employed for this study as it provides detailed three-dimensional (3D) observation as evidenced by previous studies.^9^ The fact that CBCT provides images in orthogonal planes as well as in oblique planes is an added advantage for measuring dentin thickness, canal curvature, apical transportation, and canal centering.^10^

The results obtained in our study showed less CT for the wave one single-file system as compared with the one shape system, which is in accordance with the results obtained by Saber et al^14^ and Tambe et al^15^ in a similar study on permanent teeth.

The less instrumentation time in wave one single-file system could be explained by the fact that reciprocating motion does not over engage the dentin, thus reaching the working length faster when compared with rotational motion.

The single use of endodontic instruments has been recommended recently to decrease chances of instrument separation due to fatigue and more importantly to eliminate possible cross-contamination. Because of the inability to completely clean and sterilize endodontic instruments and the possible presence of prion in human dental pulp tissue,^16^ all endodontic instruments are recommended to be of single use.

## CONCLUSION

Within the limits of this study, the new wave one NiTi primary reciprocating single-file system has proved to be a faster and safer system with less procedural errors as compared with one shape continuous rotation file. However, further investigations are needed to understand if these findings have to be attributed to the reciprocating motion, the variable section design, the M-wire technology, or a combination of these variables. Nevertheless, it could prove to be an invaluable tool for pediatric endodontic procedures.
